# Complete Genome Sequence of Escherichia coli Siphophage Shashou

**DOI:** 10.1128/MRA.01016-19

**Published:** 2019-10-03

**Authors:** Tyler Higbee, Shih-Hung Yang, Russell Moreland, Mei Liu, Jolene Ramsey

**Affiliations:** a Center for Phage Technology, Texas A&M University, College Station, Texas, USA; Queens College

## Abstract

Here, we announce the genome of the Escherichia coli 4s siphophage Shashou, which presents similarity to members of the *Guernseyvirinae* subfamily. Shashou is predicted to use a headful packaging mechanism for its 44,155-bp genome and to encode 77 proteins.

## ANNOUNCEMENT

Escherichia coli, a Gram-negative bacterium with fast growth under diverse conditions, is one of the most studied organisms on the planet (1). Primarily, E. coli inhabits the gut biome of mammals and, less frequently, soil, water, and food. Phages that infect E. coli may be useful in phage therapy against certain antibiotic-resistant pathogenic strains (2). The well-studied host provides a robust experimental system for studying the phage life cycle. This announcement reports the genome of the new E. coli 4s siphophage Shashou.

Phage Shashou was isolated from filtered (0.2-μm filter) wastewater treatment plant influent collected in College Station, Texas, by plating on Escherichia coli 4s (3). Both phage and host were grown aerobically at 37°C in Luria broth (BD), and standard soft-agar overlay methods were used (4). Genomic DNA was purified using the Promega Wizard DNA clean-up system according to the modification in the shotgun library preparation protocol given by Summer and prepared as Illumina TruSeq Nano low-throughput libraries (5). Sequencing was done with paired-end 250-bp reads using V2 500-cycle chemistry on an Illumina MiSeq system. The 844,502 total sequence reads from the index containing the phage genome were quality controlled using FastQC (www.bioinformatics.babraham.ac.uk/projects/fastqc). Reads were assembled with SPAdes v3.5.0 using default parameters, yielding a single contig of 44,155 bp for Shashou and 402.6-fold contig coverage after trimming using FastX-Toolkit v0.0.14 (http://hannonlab.cshl.edu/fastx_toolkit/) (6). Sanger sequencing of a PCR product amplified across the 5′ and 3′ contig ends (forward, 5′-GGACTCTATATGTCAAGCGGATG-3′; reverse, 5′-TGGCAGGAAATTACAGCGTAG-3′) was used to close the genome. Rho-independent termination sites were annotated from TransTermHP v2.09 (7). tRNA detection was done with ARAGORN v2.36, and gene calling was performed using GLIMMER v3.0 and MetaGeneAnnotator v1.0 (8–10). TMHMM v2.0, InterProScan v5.33-72, and BLAST v2.2.31 searches against the NCBI nonredundant (nr) and UniProtKB TrEMBL and Swiss-Prot databases with a 0.001 minimum expectation cutoff were used to predict gene function (11–14). HHPred, using the HHsuite v3.0 release at default settings with HHblits with ummiclust30_2018_08 for multiple-sequence alignment (MSA) generation and PDB_mmCIF70 for modeling, was used to predict structural similarity (15). DNA sequence similarity was calculated using progressiveMauve v2.4.0 (16). The Galaxy and Web Apollo annotation tools are hosted at the Center for Phage Technology (https://cpt.tamu.edu/galaxy-pub) (17, 18). Phage samples were stained with 2% (wt/vol) uranyl acetate and viewed using transmission electron microscopy at the Texas A&M Microscopy and Imaging Center for morphology observations (19).

Shashou is a siphophage with a 44,155-bp genome with 77 predicted protein-coding genes. It has a 50.1% G+C content and 94.4% coding density. PhageTerm predicts that Shashou uses a pac site for packaging (20).

Shashou shares 65.15% nucleotide identity and 51 proteins with *Escherichia* phage G AB-2017 (GenBank accession number KY295895), a *Guernseyvirinae* subfamily member (21). A hypothetical protein was predicted between the tail assembly chaperone (NCBI accession number QEA09424) and the tape measure protein (NCBI accession number QEA09426), with no canonical slippery sequence to induce translational frameshifting. The helicase (NCBI accession number QEA09433) in Shashou contains an intein.

### Data availability.

The genome sequence and associated data for phage Shashou were deposited under GenBank accession number MK931440, BioProject accession number PRJNA222858, SRA accession number SRR8893605, and BioSample accession number SAMN11414490.
